# Matched Molecular Pair Analysis in Short: Algorithms, Applications and Limitations

**DOI:** 10.1016/j.csbj.2016.12.003

**Published:** 2016-12-13

**Authors:** Christian Tyrchan, Emma Evertsson

**Affiliations:** Department of Medicinal Chemistry, RIA iMed, AstraZeneca R&D, Pepparedsleden 1, S-431 83 Mölndal, Sweden

## Abstract

Molecular matched pair (MMP) analysis has been used for more than 40 years within molecular design and is still an important tool to analyse potency data and other compound properties. The methods used to find matched pairs range from manual inspection, through supervised methods to unsupervised methods, which are able to find previously unknown molecular pairs. Recent publications demonstrate the value of automatic MMP analysis of publicly available bioactivity databases. The MMP concept has its limitations, but because of its easy to use and intuitive nature, it will remain one of the most important tools in the toolbox of many drug designers.

## 1 Introduction

The challenge of molecular design is the decision what to do next based on available data, medicinal chemistry expert knowledge, experience and intuition [1]. In small sets of molecules an experienced chemist can spot trends and relationships by eye. As the numbers of compounds increases, more systematical approaches are needed. Already in the early 70s, methods for systematic analysis were published e.g. the Topliss Scheme [1] or the Craig Plot [2], recommending a systematic stepwise method of building a structure–activity relationship for a chemical series. Hansch [3], Free and Wilson [4] reasoned in the 1960s that the biological activity for a set of analogues could be described by the contributions that substituents or structural elements make to the activity of a parent structure. Generally speaking these local QSAR methods try to find a correlation between structural and physicochemical descriptors towards a given endpoint [4], such as biological activity.

**Fig. 1:**
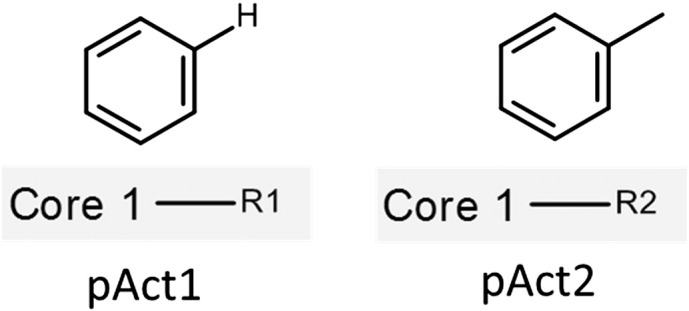
Example of a matched molecular pair (MMP).

The term Molecular Matched Pair (MMP) was coined in 2004 by Kenny and Sadowski [5], for a special case of QSAR; now a widely used concept throughout drug design processes. In the most common situation, MMP describes a pair of compounds that differ structurally at a single site through a well-defined transformation (see Fig. 1) that is associated with a relative change in a property value. The correlation between the structural change and the property change is used in rationalizing observed structure–property-relationships (SPR) and compound optimization. Several different applications for MMP analysis originating from industry or academia have been developed and published, highlighting its importance. Among others these include: Drug-Guru [6], [7], Buy me Grease [7], WizePairZ [8], T-Analyse and T-Morph [9], VAMMPIRE [10] as well as the Hussain-Rea MMP algorithm [11] (Table 1). The MMP concept has been further developed into Matched Pair Series [12], [13] or Matched Molecular Series (MMS) [14] to describe a set of compounds (not only a pair) differing by only a single chemical transformation.

Recently an extension of the MMP concept towards biopharmaceutical applications was published, using macromolecular sequence data to predict the effect of single amino acid substitutions on property optimization [15].

Besides supporting hypothesis development and testing, an important application of MMP is in the detection of outliers, namely a pair of compounds that show a step change in a property; a so called activity cliff. These compounds are often the most interesting to study in the design of compounds targeting improvement of the property showing this change [16], [17]. An inherently difficult problem to detect these activity cliffs is confounded by experimental uncertainties in the measured properties, since they are a function of the chemical space representation [18]. One systematic approach to the detection of activity cliffs and determination of their depth uses support vector regression [19]. Not only can different chemical space representations lead to significant changes in the nature of these activity cliffs, but even simple atomic variations can cause dramatic effects on important complex endpoints in medicinal chemistry; dose to man prediction, potency, clearance, solubility and permeability to name a few [18], [20]. If the structural change (R group) is small and the scaffold in a chemical series is conserved, the MMP represents a relevant and easy to interpret chemical space representation. The MMP approach can further be extended to systematically analyse non-additivity in a structure property relationship (SPR) series [21].

## 2 Application and Limitation

The assumption that the effect of chemical substitution can be generalized, is inherently assumed in all QSAR methods, including the MMP approach, successfully highlighted by the work of Lipinski et al. who correlated physicochemical properties to oral bioavailability [22]. With the increasing availability of public databases containing millions of structure–activity-relationship (SAR) [23], [24] or SPR data, multiple papers have been published applying MMP concept to: ADME [25], [26], bioisosterism [9], [27], [28], aqueous solubility [29], [30], [31], [32], plasma protein binding [29], [30], oral exposure [29], logD [8], [30], [32], potency [8], [9], [27], [31], [33], intrinsic clearance [7], [34], herG and P450 metabolism [29], [32], [34], in vitro UGT (Uridine 5′-diphospho-glucuronosyltransferase) glucuronidation clearance [35], half-life [31], selectivity against off-targets [31], impact of N- and O-methylation on aqueous solubility and lipophilicity [36] or mode of action; [31] the analysis differing only in the MMP algorithm used.

In two relatively recent publications [31], [37] an apparently simple MMP transformation of CH → C-CH3 is analysed in greater detail and highlights some general limitations and drawbacks of using the MMP concept prospectively in drug design. The methyl group is a commonly occurring carbon fragment in small-molecule drugs and can modulate both the biological and physical properties of a molecule. Two literature analysis of > 2000 cases of methylation revealed that an activity boost of a factor of 10 or more is found with an approximate 8% frequency, and the probability of achieving a 100-fold boost is less than 1% [33]. However, the distribution of potency changes in respect to the MMP is often nearly symmetrical and centred at or near zero resulting in a similar likelihood of causing potency gains or losses. A consistent bias of specific substituents towards improved potency could not be observed. Nevertheless an understanding of these rare events affecting the binding potency by improving the IC_50_ value of a compound by more than 100-fold would provide great value in prospective affinity optimisation. From logD measurements the free energy of binding can be estimated for this specific transformation to be about 0.8 kcal/mol [31]. This corresponds to an approximate 3.5-fold boost in potency from methylation based on partitioning effect alone. A more empirical evaluation of literature examples by Jorgensen and co-workers suggested that a single methyl group might actually boost potency approximately 10-fold if the new methyl group sits optimally in a hydrophobic pocket of the active site [37]. Further they found that methyl substitutions ortho to an aryl ring can be particularly effective at modulating activity due to the induction of a conformational change. Coupling the conformational gain with the correct placing of the methyl group in a hydrophobic pocket of the protein might therefore result in the greatest improvements of activity. It must be emphasized that this does not explain all observations of a > 100-fold boost in potency and the reasons for this increase are not necessarily straightforward to rationalize.

Besides the rationalization of the observed property change in a structural context, the prospective use of MMP is further hampered by several additional limitations. These are discussed in various publications [12], [38] and include: the problem of generalized relationships like global versus local effects, molecular context, database, time and data set dependencies and definition of the relevance of observed difference (e.g. cut-off at 2 fold change). The interpretation of the relative property value change in the MMP analysis is also dependent on the experimental error [39], [40]. Commonly authors use e.g. Z-Scores to define the significance of the potency difference [9], the mean, standard deviation and standard error [33], [41] or a specific value cut-off [42], [43]. By assuming different experimental errors Kramer et al. showed how the minimum number of pairs necessary to achieve significance can be calculated, as they explained the difference between statistical significance and effect size estimation [21], [44]. Relevant experimental errors for public and industry SAR databases are nowadays published [21], [39].

**Fig. 2:**
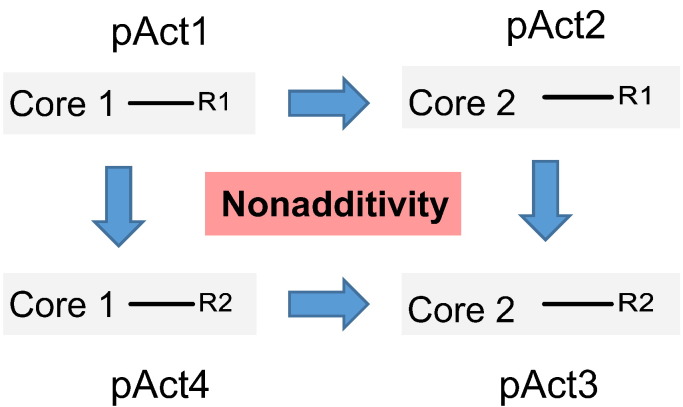
Matched Square Pairs cycle to determine non-additivity in a SAR analysis.

Finally, MMP analysis as a linear substituent contribution model generally assumes additivity and thus do not work in cases of non-additivity [21]. Matched Square Pairs, an extension of MMP analysis, can allow a judgement of the quality of generalized relationships. By looking at squares (pairs of matched-pairs) it is possible to check for non-additivity and for outliers. The matched square shows four transforms, which could involve a change of the core and two R-groups or one core and three R-groups as shown in Fig. 2. Non-additivity is calculated as (pAct3-pAct4)-(pAct2-pAct1) [21] and indicates if there is an apparent non-linear SAR for a subset of compounds. This in turn could lead to new binding or interaction hypothesis e.g. conformational changes or restrictions leading to different binding mode or protein dynamics.

## 3 MMP Algorithms

In principle all published MMP algorithms can be defined as supervised or non-supervised methods. In supervised methods the chemical transformation that generates the MMP is predefined, while in the unsupervised methods an algorithm is used to find all possible pairs in a set of compounds, mainly using maximum common substructure (MCS) or fragmentation approaches [38], [45]. The advantage with supervised methods lies within the precise control of the definition of the MMP to address a particular question [7], [29], [34]. On the other hand, these methods cannot find new and surprising MMPs in the way that unsupervised methods can.

In the 90s Van Drie and coworkers defined structure activity landscape index as:

$${\mathrm{SALI}}_{i,j}=\frac{\left|A_{i}-A_{j}\right|}{1-\mathrm{sim}\left(i,j\right)}$$

where *A*_*i*_ and *A*_*j*_ are the activities of the *i*th and the *j*th molecules, and sim(*i*, *j*) is the similarity coefficient between the two molecules [16] in an approach to find compounds with small structural differences and large difference in properties. Guha further extended the structure activity landscape to predict compounds with ability to have activity cliffs [46]. Later, Wassermann et al. [27] analysed matched molecular pairs, generated by a modified reimplementation of the Hussain-Rea algorithm, in respect to their ability to introduce activity cliffs using public domain compound data. Approximately 250 nonredundant substitutions were identified with tendency to display activity cliffs. A definition of activity cliff is given and distribution of MMPs is shown. Hu et al. [43] further looked at these activity cliffs with substructures (MMP) in contrast to similarity searches and could identify more relevant cases with MMP. Sheridan et al. [9] screened for the most common chemical replacements in a large collection of drug-like molecules from the MDL Drug Data Report. Different treatments of replacements in rings are implemented and used to identify potential bioisosteres. A maximum common substructure (MCS) approach was used to define the MMP, based on a clique detection method and one single replacement side. Similarly, Haubertin and Bruneau [30] used about 9000 predefined functional groups to analyse the effects on a corporate compound deck in respect to various compound properties. For MMP creation they used a RECAP fragmentation algorithm which identified one of the predefined groups. Hajduk and Sauer [33] looked at the influence of common chemical substitutions on ligand potency. Overall 127 different chemical changes were compared and shifts analysed. MMPs were identified by a pairwise comparison with the findsub routine from Daylight, which looks at terminal or side chain groups only. The analysed database consisted of 84,000 compounds from lead optimisation of more than 30 different targets. They couldn't find a substituent which perfectly biased the result in an always gain or loss, the distribution was normal and nearly symmetrical centred at or near 0, with a potency change probability of 10 fold gain around ~ 8.5%, 100 fold less than 1%; similar to the findings of Schönherr et al. [31].

Raymond et al. [47] used a MCS based MMP algorithm to identify chemical changes within a collection of 2,7 million compounds and discussed statistical relevance of these modifications. Also published in 2009, Gleeson et al. [26] used a partial supervised MMP algorithm by defining a given transformation described by a substructure combined with a retrieval algorithm to find all transformations of this substructure to derive ADMET rules of thumb. Hussain and Rea [11], [34] introduced in 2010 an efficient fragmentation algorithm to systematically extract all MMPs from a given compound data set. They perform single, double and triple cuts at all bonds, without breaking ring bonds. Some retrieval problems are discussed e.g. alcohol to amide would retrieve compounds where a carboxylic acid has been substituted with an amide.

Dossetter [34] analysed groups in a similar fashion as Topliss [1] in applying a statistical analysis to in vitro human microsomal metabolic stability data for small phenyl group substituents using AstraZeneca inhouse proprietary software, ThricePairs. ThricePairs uses SMARTS patterns to specify substructures. This was later followed by WizePairZ [8], described in 2010 by Warner et al. as a tool which can automatically detect and identify matched molecular pairs and encoded them in SMIRKS reaction notation. In essence, it is a MCS method which captures different levels of the local single site environment similar to the approach of Sheridan. Using SMIRK definitions Ritchie et al. [48] looked at the replacement of mono-substituted benzene ring with aromatic or aliphatic heterocycle MMPs and the effect on nine ADME properties.

Weber et al. [10] published in 2013 a strategy to relate the substitution effect within matched molecular pairs to the atom environment within the cocrystallized protein–ligand complex with the aim to predict ligand binding from extrapolation of the effect of the substitution with the molecular environment taken into account [49].

In 2014 Leon et al. [50] introduced a method to automatically generate synthetically accessible MMPs by applying reaction rules following the retrosynthetic combinatorial analysis procedure (RECAP) with the aim to generate more chemically interpretable and accessible pairs. A library of more than 92,000 RECAP-MMPs was generated from public domain compounds active against 435 different targets exclusively utilizing high-confidence activity data.

MMP has also lately been coupled to network analysis of composition and topology to be able to predict potency as in QSAR type approaches [51]. Similarly, Ghosh et al. [52] recently published a study where pairs from matched molecular series (MMS) were coupled to SAR information creating a database of MMS with SAR characteristics to be used in future medicinal chemistry work. They applied the fragmentation technique of Hussain and Rea to a set of 48,000 bioactive compounds.

## 4 Conclusion

MMP has been used for more than 40 years within molecular design and is still an important tool to analyse potency data and many other compound properties. The methods used to identify matched pairs range from manual inspection, through supervised methods to unsupervised methods. The MMP framework allows one to study numerous properties (most commonly binding affinity or potency) and to rationalize the design of the next compound to make within a series. Despite its usefulness several limitations have to be considered, including: the representation of molecular structures, selection of the most appropriate algorithm for the task, and the statistical analysis method applied to the data to ensure that the found property difference is indeed relevant. This is especially important in respect to identifying activity cliffs, where a small chemical change relates to a large change in potency or another property of interest. These activity cliffs are often the most important MMPs to study within a lead series. In contrast to traditional SAR analysis, where similar compounds are assumed to have similar properties, activity cliffs describe the substitution pattern with the most impact upon a small structural change.

Because of the intuitive nature of the MMP concept through connecting small structural changes to a property change, and the maturity of its framework and simplicity of use, it will remain one of the most important tools in the toolbox of a drug designer.

## Figures and Tables

**Table 1:** Classified MMP algorithms.

Non-supervised methods			
	R. Guha (2012) [46]	BCI structural fingerprints, CDK 1024-bit path fingerprint	2016
	Fuchs et al. (2015) [15]	Sequence alignment for peptide MMP^a^	2015
	T.J. Ritchie et al. (2015) [36]	HRF^b^	2015
	Matsy (2014) [13]	HRF^b^	2014
	VAMMPIRE (2013, 2014) [10], [49]	MCS and HRF^b^	2013
	C.E. Keefer et al. (2011) [25]	Modified HRF^b^ (Pairfinder)	2011
	J. Bajorath et al. (2010–2016) [12], [14], [19], [27], [43], [50], [51], [52]	HRF^b^, modified HRF^b^, RECAP^c^ fragmentation	2010
	J. Hussain et al. (2010) [11]	Hussain and Rea fragmentation (HRF^b^)	2010
	L. Cururull-Sanchez (2010) [35]	ECFP6 fingerprints with sub-structure search	2010
	Papadatos et al. (2010) [42]	dt_commonsubstruct and findsub routine from Daylight and HRF^b^	2010
	WizePairs (2010) [8]	MCS^d^ and SMIRKS^e^	2010
	Raymond et al. (2009) [47]	MSM^f^ rule framework based on MCS^d^	2009
	R. Sheridan et al. (2002, 2006) [9], [28]	Similarity and MCS^d^ method (T-Analyse)	2008

Supervised methods	ThricePairs (2010) [34]	Defined transformations, SMARTS^g^	
	Gleeson et al. (2009) [26]	Substructure Search	2010
	Buy me Grease (2009, 2010) [7], [35]	Defined transformations, RXN^h^ format	2009
	P.J. Hajduk et al. (2008) [33]	Findsub routine from Daylight and defined transformations, SMIRKS^e^	2009
	D.Y. Haubertin et al. (2007) [30]	RECAP^c^ method	2008
	Drug Guru (2006) [6]	Defined transformations, SMIRKS^e^	2007
	N.T. Southall et al. (2006) [53]	Topological torsion similarity and MCS^d^	2006
	A. G. Leach et al. (2006) [29]	Defined transformations, SMARTS^g^ (Leatherface)	2006
	T.J. Ritchie (2016) [48].	SMIRKS^e^	
			2006

a MMP: Molecular Matched Pair.

b HRF: Hussain and Rea fragmentation.

c RECAP: Retrosynthetic Combinatorial Analysis Procedure.

d MCS: Maximum common substructure.

e SMIRKS: SMILES reaction specification.

f MSM: molecular substructure modification.

g SMARTS: SMiles ARbitrary Target Specification.

h RXN: MDL Molfile Reaction Format.
